# Analysis of Selected Body Composition Parameters and Ergonomic Safety among Professionally Active Nurses in Poland: A Preliminary Prospective Monocentric and Observational Study

**DOI:** 10.1155/2020/9212587

**Published:** 2020-08-03

**Authors:** Anna Kołcz, Martyna Baran, Karolina Walewicz, Małgorzata Paprocka-Borowicz, Joanna Rosińczuk

**Affiliations:** ^1^Laboratory of Ergonomics and Biomedical Monitoring, Wroclaw Medical University, Grunwaldzka 2, 50-355, Wroclaw, Poland; ^2^Department of Physiotherapy, Wroclaw Medical University, Grunwaldzka 2, 50-355 Wroclaw, Poland; ^3^Department of Neurological Rehabilitation, Regional Specialized Hospital in Wroclaw, Poświęcka 8, 51-128 Wroclaw, Poland; ^4^Institute of Health Sciences, University of Opole, 45-060 Opole, Poland; ^5^Department of Nervous System Diseases, Wroclaw Medical University, Bartla 5, 51-618 Wroclaw, Poland

## Abstract

Nurses consist of an occupational group that is particularly exposed to harmful work-related factors such as prolonged working hours, severe stress, fatigue, and excessive strain on the musculoskeletal system. According to nurses, the limitation of the application of ergonomic principles of work may contribute to the occurrence of numerous dangerous behaviors, improper eating habits, or deficiency of systematic physical activity. The most common consequences are nutritional disorders and musculoskeletal system dysfunctions. This prospective observational study was aimed at evaluating selected parameters of the body composition of professionally active nurses and at determining work-related risks during nursing activities. The study group consisted of 37 active nurses (38.38 ± 11.33 years). The research tool was a device for bioelectrical impedance analysis (BIA). A questionnaire designed by the authors was also implemented, which covered ergonomic principles, musculoskeletal injuries, and nutritional habits. In the present study, it was shown that all average values of the tested nurses' body composition parameters were within the normal range. The majority of respondents (97.3%) reached a high level of body water. A statistically significant correlation was found between the knowledge of the workplace ergonomic principles and body mass index. In conclusion, musculoskeletal pain and lack of implementation of ergonomic behaviors are a significant problem among nurses, which may be the cause of overweight or obesity in this occupational group.

## 1. Introduction

Contemporary literature [1–4] indicates unhealthy aspects of nurses' lifestyles. This is affected by a wide range of occupational hazards such as the specificity of work, exposure to long-term stress, overwork, shift work, improper dietary habits, insufficient daily physical activity, and frequent pain resulting from noncompliance with ergonomic principles at work [5–7].

As one of the biggest groups of health care professionals, nurses are particularly exposed to risk factors that can lead to overweight and obesity, such as long-term stress [8]. More than 60% of nurses declare that they are exposed to stress at work [9], and they suffer from sleep disturbances resulting from occupational stress [10]. Current data indicate that every second, registered nurses experience professional burnout [11], and the data also assessed the level of stress as high. This is directly linked to low salary levels, overwork, and understaffing [12].

The prevalence of pain in the musculoskeletal system among nurses in Poland was indicated by the results of a study by Mikiciuk et al. [13], who determined that 81% of female hospital personnel in Poland experienced the pain of the lumbar and 53% the pain in the cervical region of the spine. The lumbar pain was most often triggered during lifting and the pain in the cervical region during sitting. Also, 44% of nurses took analgesics in case of lumbar and 30% in the case of cervical pain. Similar results were obtained by other researchers [14–17].

The occupational group of nurses is particularly exposed to risk factors that can lead to overweight and obesity, such as long-term stress [8]. More than 60% of nurses declare that they are exposed to stress at work [9], and they suffer from sleep disturbances resulting from occupational stress [10]. Current data indicate that every second, registered nurses experience professional burnout [11], and the data also assessed the level of stress as high. This is directly linked to low salary levels, overwork, and understaffing [12].

High cortisol levels increase appetite and thus are a key factor in so-called stress eating [18]. In addition to increased appetite, prolonged stress can lead to a seemingly increased sense of pleasure, so that eating is identified with the sense of happiness and is taken more often and/or in larger quantities. Stress also disrupts the concentration of leptin in the body, increasing the threshold of food satisfaction and increasing the number of calories taken to achieve the feeling of satiety [19].

The professional group of nurses is also exposed to prolonged working hours, which in turn may ultimately limit the time spent on physical activity and make it impossible to prepare meals. Moreover, improper eating habits, caused by snacking between meals and not following the rules of healthy eating, were pointed out [20]. Every second nurse admits to drinking about 250-500 ml of water a day, which is definitely below the recommended values [21].

Shift work is a significant contributor to poor dietary choices. A study by Bilski [22] on 241 nurses showed that during the night shift, they most often consume cold meals and drink statistically more coffee than in nonshift work. Not adhering to the principles of healthy eating and limiting physical activity is already noticeable in nursing students. The study by Walentukiewicz et al. [23] showed that future nurses achieved significantly lower results in three categories of health behaviors, i.e., healthy eating habits, positive psychological attitudes, and health practices. Similarly alarming results concerning female nursing students were obtained by Blake et al. [24].

In terms of the working conditions, the nurses' lifestyle is also affected by frequent musculoskeletal pain, which negatively affects undertaking physical activity during their leisure time [25]. Work-related musculoskeletal dysfunctions are the most common condition for occupational health among nurses worldwide [26]. Bilski and Sykutera [27] noted that the majority (73.23%) of Polish nurses suffered from musculoskeletal pain, especially in the lumbar (51.64%) and the cervical (14.08%) region of the spine.

These consequences mentioned above are well known as harmful to nurses and are considered as nursing-specific workplace stressors affecting their everyday practice as well as diminishing the quality of nursing care delivered to patients [28, 29]. Poor adherence to work-related ergonomic principles during nursing activities is also an increasingly common problem [30], which is also commonly neglected by nurses [31]. These occupational factors can have a negative effect on eating habits and lead to adverse changes in body composition in the professional group of nurses, but so far, there is limited research on this subject [32]. Moreover, it should be emphasized that using bioelectrical impedance methods for the body composition analysis among nurses is surprisingly understudied.

Therefore, the primary aim of this study was to analyze selected body composition parameters such as body mass index (BMI), body fat (BF), muscle mass (MM), metabolic age (MA), visceral fat (VF), and total body water (TBW) among nurses. The secondary aim was to determine work-related risks during nursing activities.

## 2. Material and Methods

### 2.1. Settings, Design, and Participants

This prospective and observational study involved a group of 37 nurses who were students of the part-time master's nursing program at the Wroclaw Medical University in Poland. The study was conducted at the Department of Physiotherapy of the Wroclaw Medical University in Poland from May 2017 to December 2017. The STROBE (Strengthening the Reporting of Observational Studies in Epidemiology) guidelines were followed [33].

### 2.2. Ethical Considerations

The study was approved by the independent Bioethics Committee of the Wroclaw Medical University in Poland (no. KB–205/2018). The study was completely anonymous, and each of the nurses surveyed gave voluntary and written consent to participate in the study. The study was conducted according to the Declaration of Helsinki and Good Clinical Practice guidelines [34].

### 2.3. Qualification Criteria

Inclusion criteria were (1) status of the professionally active nurse, (2) lack of contraindications to measurement with bioelectric impedance, (3) lack of diagnosed chronic systemic disease or metabolic disorders, (4) lack of lower limbs swelling due to venous or lymphatic insufficiency, and (5) voluntary written consent to participate in the study.

In turn, the exclusion criteria comprised (1) lack of the status of professionally active nurse, (2) presence of contraindications to bioelectrical impedance measures such as pacemaker and other electronic or metal implants, (3) pregnancy or menstruation, (4) failure to follow the recommendations before the tests (eating meals less than 4 hours before the test, taking physical activity, and/or drinking alcohol less than 12 hours before the test), and (5) lack of written consent to participate in the study.

### 2.4. Measurement Tools

#### 2.4.1. Survey Method

In the first stage of the study, nurses were asked to fill in a questionnaire developed by the authors for the purpose of this study. This was a simple survey which included questions about nurses' age, length of seniority, number of working hours per week, number of hours per day spent while standing, level of knowledge and implementation of ergonomic principles, musculoskeletal injuries and pain, ways to deal with pain, and any overweight or obesity episodes that may have occurred in the past.

#### 2.4.2. Bioelectrical Impedance

Selected body composition parameters were analyzed using the TANITA MC-780 S MA analyzer (TANITA Corporation, Tokyo, Japan), which uses the phenomenon of bioelectrical impedance analysis (BIA). The BIA device was connected to a computer equipped with the TANITA GMON MDD Professional software (Medizin & Service GmbH, Chemnitz, Germany). The BIA method is one of the most frequently used methods for body composition analysis. It is a noninvasive, safe, and applicable to people of all ages [35, 36] and is widely used in both medical and dietary offices, for practical and research purposes. Also, this technique has very high repeatability—the reliability factor test-retest for a four-electrode system is 99%.

**Figure 1:**
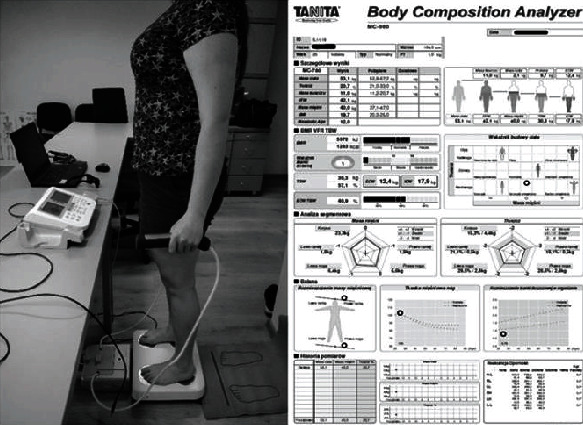
Participant during measurement with the use of the TANITA device.

The BIA enables the evaluation of such indicators (% and kg) as body fat (BF), fat-free mass (FFM), skeletal muscle mass (SMM), basal metabolic rate (BMR), metabolic age (MA), visceral fat (VF), total body water (TBW), extracellular water (ECW), intracellular water (ICW), total bone mineral (TBM), and segmental BF [37]. An example of BIA examination and test report presenting the results is shown in Figure 1.

### 2.5. Statistical Analysis

The obtained data was encoded and transferred to MS Office Excel 2017 and then subjected to statistical analysis using Statistica version 13.3 (TIBCO Inc., Tulsa, USA). The basic description of quantitative variables such as mean (M), median (Me), standard deviation (SD), maximum (max), and minimum (min) was made during the preparation of the results. Qualitative variables (nominal and ranged) are described in percentages and numbers. The chi-squared test was used to compare qualitative variables. The *t*-test or Mann-Whitney *U*-test was used to compare quantitative variables. The correlation analysis of the studied variables was evaluated using the Spearman correlation coefficient (rho). The level of statistical significance was set at *p* < 0.05.

Sample size analysis was performed in Statistica 13 (TIBCO Inc., Tulsa, United States). The *α* level was set at 0.05 with a confidence interval of 95% and the power of the test at 0.8. It also assumed no correlation of evaluated variables and adopted a 2-sided null hypothesis. On the basis of the parameters, the estimated sample size has been obtained equal to 37 people in the study group.

## 3. Results

### 3.1. Participant Characteristics

The study included 37 nurses with a mean age of 38.38 ± 11.33 years. The largest group of nurses, i.e., 54%, had more than 20 years of seniority; the second largest number of nurses (24%) had less than two years of seniority. The study group also differed in terms of the number of working hours per week, with the highest number of nurses working 30-40 hours per week and 40-50 hours per week (40.5%). Also, 40.5% of the nurses spent more than 8 hours and between 5 and 8 hours a day in standing position. Detailed characteristics of the study group are presented in Table 1.

The average height of the nurses was 165.8 cm, while the average body weight was 66.4 kg that gives an average BMI of 24.1 kg/m^2^, which was within the upper ranges according to the World Health Organization (WHO) classification. The normal range of BMI (18.5–24.9 kg/m^2^) was observed in 26 of the respondents (70.3%), with BMI indicating overweight or obesity (≥25.0 kg/m^2^) in 10 respondents (27%) and BMI indicating underweight (<18.5 kg/m^2^) in only one person (2.7%). Detailed results are presented in Tables 2 and 3.

### 3.2. Body Composition

The average percentage of BF in the study group was 29.2%, which is also in line with the TANITA norms (up to 33% for women between 20 and 39 years old and up to 34% for women between 40 and 59 years old) as well as those of the American Council on Exercise (up to 31% for adult women). The percentage of BF within the normal ranges corresponding to respondents' age was found in 29 nurses (78.5%), while the result indicating overweight or obesity was found in 8 nurses (21.6%) (Tables 2 and 3).

The percentage of SMM content of the studied nurses was 67.2%. The TANITA analyzer indicates the SMM standard's scope individually, matching it to the age, sex, weight, and height of the examined person. As a result, 32 persons (87%) achieved SMM values within the standard and five persons (13%) exceeded the standard. None of the subjects surveyed showed a too low SMM parameter (Tables 2 and 3).

The MA of the studied nurses was 33.5 years old, which, compared to the metric age, gives a result lower than five years. The MA of individual respondents confirms this: 73% of them are older in terms of the calendar than their MA indicates it to be. More than a quarter of the respondents reached the MA parameter higher than the calendar age (Tables 2 and 3).

The average percentage of TBW in the study group was 50.6%. This result is within the range of the standard (45-60% for adult women). The vast majority of the examined group, i.e., almost 92%, achieved the result within the norm. None of the nurses tested showed TBW above the standard (Tables 2 and 3).

The ECW/TBW ratio was 43.2% on average, with a median of 43.2%. This is within the normal ranges specified by TANITA (up to 45%) but indicates a value between 35% and 40% and may indicate dehydration. Only one person reached ECW/TBW within the recommended range, so a slight degree of dehydration can be suspected in most studied nurses (Tables 2 and 3).

According to TANITA, values between 1 and 12 are standard, so the observed results were in the lower range. None of the nurses reached the value exceeding the norms, although several results were set at the upper limit. The majority of nurses reached the values of 1 and 5 (8 for each of these values). Only four nurses achieved a result equal to or greater than 8, which confirms that the vast majority of the study group showed low VF values (Tables 2 and 3).

### 3.3. Workplace Ergonomics

Among the surveyed nurses, as many as 12 admitted that they did not have any knowledge of the principles of ergonomics in their profession. However, some of them (after familiarizing themselves with some principles of ergonomics in the course of the study) stated that they sometimes apply them but entirely unintentionally. What is more, 24.3% of respondents admitted that they never apply the principles of workplace ergonomics.

A statistically significant correlation between the knowledge of the principles of workplace ergonomics and the BMI values of the studied nurses was shown (rho = −0.38, *t* = 0.02, and *p* = 0.02). The average value of BMI in nurses who did not have any knowledge about principles was more than 3 kg/m^2^ higher than that in nurses who have such knowledge at work indicating overweight. The percentage of overweight or obese nurses was more than twice as high in the group who did not know the principles of workplace ergonomics.

The percentage of overweight nurses was 44.4%, which was more than twice higher compared to nurses who adhere to the principles of ergonomics at work.

The study group was also asked about musculoskeletal pain that occurred during the last 12 months before the study. Only one person did not mention the condition in any of the nine areas covered by the question. The highest number of respondents complained about back pain (lumbosacral in 70.3%, cervical in 62.2%, and thoracic in 56.8%). Ailments in the lumbosacral region accounted for slightly more than a fifth of all reported ailments (Table 4).

**Figure 2:**
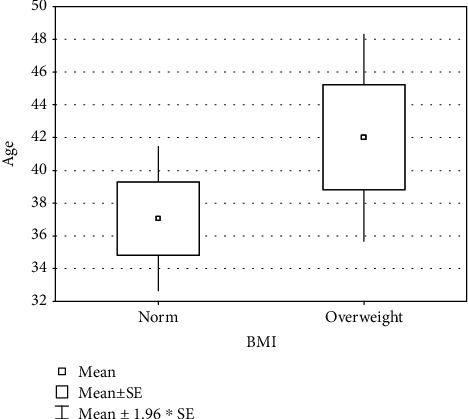
Comparison of the participants' age depending on the BMI values.

BMI values in the study group differed depending on the age of the study subjects. The average age of people with a BMI value within the norm was 37 years. In the case of people with a BMI value indicating overweight or obesity, the average age was five years higher (Figure 2).

Parameters such as length of service, weekly working hours, and time spent in standing position at work was also compared to overweight in the past. Some of the subjects were not overweight or obese during the study but admitted that this happened previously. A trend was shown that the incidence of overweight in the past was affected by length of service. The higher the length of service, the slightly increased the risk of overweight in the past. However, the observation was not statistically significant (*p* = 0.24).

## 4. Discussion

The results of the present study suggest that the mean body composition parameters in the study group of nurses were within the range of the norm. Parameters such as BMI, BF, and ECW/TBW were in the upper ranges of norms. Among the nurses studied, the criteria of overweight or obesity concerning BMI met 27.03%, which is quite a large percentage. However, this value is much lower than the rate of overweight and obese people in Polish society. According to Stepaniak et al. [38], the prevalence of overweight is 43.2% in men and 30.5% in women, while abdominal obesity was noted in 32.2% of men and 45.7% of women.

Pawloski and Davidson [39] assessed body composition changes as a specific indicator of obesity among a group of female nursing students with mean age of 29.29 ± 7.96 years. They found that participants had a mean BMI of 24.89, and the mean body fat percentage was 35.00% when suggested percent body fat standards for an adult male over 25% indicate obesity. Also, Chin et al. [40] in their cross-sectional study among 394 nurses with mean age of 48.4 ± 12.1 years demonstrated that 31.1% of respondents were overweight and 17.6% were obese. Moreover, overweight/obesity (BMI ≥ 25 kg/m^2^) was significantly more common among nurse managers and nurses who worked full time or worked ≥40 h per week which confirms correlation with occupational factors.

The research conducted among Polish nursing students showed that body weight values exceeding the norm concerned 20% of people. In the same study, 5% of female students found that their body weight values were too low. In our research, we found a higher percentage of overweight and a lower percentage of underweight nurses. However, this is understandable due to the age of the study group. In the study by Walentukiewicz et al. [23], the average age was 19.1 years, while in our study, it was 38.4 years.

In our study, the average BMI value of the study group with age range 22-43 years was 24.1 kg/m^2^. It was similar to results by Przeor and Goluch-Koniuszy [41] in the study group of nurses aged 31-50 years where the average BMI was 24.9 kg/m^2^. This result is slightly higher than that in our study and may result from the age difference between these study groups which indicates that better BMI characterizes younger nurses.

Very few studies use parameters other than BMI to assess overweight or obesity. There are even fewer studies that involve a group of professional nurses. There is a justified difficulty in comparing our results with those obtained by other authors in terms of parameters such as fat tissue accumulation or hydration (TBW).

One of the Polish studies by Kaska [42] among 210 nursing students showed that both the average BMI and BF were lower than in our study. The percentage of overweight students measured with BMI was also lower than what was observed in our study (22.4% vs. 27%). However, the rate of overweight students assessed with BF turned out to be more than 10% higher than that in our study. This seems to be a surprising result given the age of the respondents. The difference in hydration between the students from the study by Kaska [42] and the nurses from our study also seems surprising—it turns out that the students achieved the average TBW index by almost 20% lower than the working nurses.

One of the most unfavorable results in the study group is the ECW/TBW values achieved. At the same time, only one person reached the recommended value below 40%, which means that 97.3% of the study group is more exposed to dehydration. As many as 16.2% of the examined nurses achieved results above 45%, which indicates that they were dehydrated.

A well predictive result is the low VF values achieved by the studied nurses. Even people who had body composition parameters indicating obesity achieved normal results. This may indicate that nurses as a professional group are not particularly exposed to high levels of VF. This is a positive result because, as well known, an increased VF level is an independent risk factor for sudden death and many systemic diseases [43].

The results obtained in our study on musculoskeletal pain among nurses and ways of coping with them do not differ significantly from the results obtained by other authors. In our study, as well as in those by Bilski and Sykutera [27] and Mikiciuk et al. [13], it was shown that work-related musculoskeletal pain which concerns nurses most often is in the lumbar and cervical regions of the spine. The same situation is observed for the ways of coping with pain by nurses. Our study has shown that the majority (37.8%) of the nurses examined cope with musculoskeletal pain by taking oral analgesics.

Our research showed a statistically significant correlation between BMI value and nurses' knowledge of work ergonomics, as well as a tendency to achieve higher BMI values among people who did adhere to ergonomic principles. As we mentioned before, this may be due to noncompliance with ergonomic principles on the occurrence of musculoskeletal pain observed by other authors.

Mikiciuk et al. [13] have demonstrated a correlation between many work activities performed in a nonergonomic way (e.g., working in an inclined position or carrying heavy patients) with pain problems in nurses. Bilski and Sykutera [27] showed similar correlations, although this was more concerned with the transfer of patients lying down and the presence of ergonomic solutions in the wards (lifting equipment). Tantawy et al. [44] showed a correlation between the occurrence of musculoskeletal pain and higher BMI values.

Nam et al. [45] performed their study on the sample of 454 nurses (mean age 49.6 ± 13.1 years), and they reported that the mean BMI was 25.6 kg/m^2^, and 47.7% were overweight or obese. What is more, the work-related musculoskeletal overload was observed: low back symptoms were most frequent (61.7%), neck pain (48.5%), shoulder pain (41.9%), and wrist/hand pain (41.6%) were also common symptoms.

Another statistically significant correlation shown in our research was between work seniority and BMI. However, it is most probably related to differences in age and aging changes. It is a fact that the level of BF begins to increase between 20 and 30 years of age and reaches its maximum, usually at the age of 50-60. This phenomenon is mainly related to the reduction of total energy expenditure while maintaining the same or a small change in the diet's energy level (hormonal changes play an important role, especially in women) [46].

Even though our study did not show a statistically significant relationship between working hours per week and the BMI and the occurrence of overweight or obesity, it could be observed that the average BMI value was the highest in the group of nurses working more extended number of hours. Such a result can determine many different factors—one of them may be stress due to overwork [47]. Previous studies pointed out the importance of shift work on the level of stress and diet of nurses and even to the value of their BMI [48].

### 4.1. Strengths and Limitations

There are several strengths of this paper which should emphasized. In light of the current demographic and economic situation of the health care system in many European and worldwide countries, there is a need to extend the working lives of health care workers, especially nurses. This study indicates that the implementation of appropriate health monitoring solutions, including weight composition assessment and ergonomic behavioral control which are important. Overweight and obesity are among the most common health problems in the population of women aged 55-65, who are becoming the most numerous age group working in the health care system. In the current favorable demographic situation, with the lack of generational substitutability and an aging population of nursing personnel, the most important task seems to be to monitor the ability to work and the possible risks of noncompliance with ergonomic principles.

This study has some potential methodological limitations to be mentioned. First of all, our study has a preliminary design with a sample of 37 subjects involved, and it should be continued in a larger and more representative group of nurses. Unfortunately, our research did not consider factors such as stress and diet, which would allow a broader context to be presented for potential disturbances of body composition parameters. The same applies to the level of physical activity undertaken by nurses in their free time and its effect on the body composition parameters. In our study, we asked about physical activity only in the context of how to deal with pain, and it was not correlated with the level of BMI. The issues mentioned above should be considered in future studies, as should including the participation of a control group of persons who are not part of the professional nurses' group.

### 4.2. Practical Implications

Based on our study results, there are some implications for clinical practice that might be considered by nurses and their nursing managers. More attention should be paid to the body composition parameters in the context of ergonomic principles among nurses. Nurses should be routinely and systematically screened for their body composition and should be controlled regarding their level of hydration, especially in those with abnormal body mass (overweight, obesity). The BIA measurements may be a useful and simple way to assure a proper control of the body composition.

Systematic observation and analysis of body mass composition, as one of the elements of health monitoring, increase awareness of the health risk resulting from perimenopausal changes. The assessment of body weight alone becomes insufficient because changes in body weight components, especially the increase in fat mass in the total body weight, fat tissue around internal organs, and the restriction of hydration with age, cause clinically significant metabolic disorders. Particularly dangerous is the excessive concentration of visceral fat, which results in visceral obesity, insulin resistance, hypertension, ischemic heart disease, and type 2 diabetes. Therefore, from a clinical and practical point of view, conducting systematic monitoring of medical personnel's body composition can exaggerate the development of many diseases and effectively keep healthy staff at work.

## 5. Conclusions

We demonstrated that most of the body composition parameters among professionally active nurses are within the norm limits. Only one expectation was observed in this matter, and it was a disturbing degree of body hydration. Work-related musculoskeletal pain and a lack of knowledge in implementing ergonomic principles in everyday nursing practice are significant problems, which can contribute to the occurrence of overweight and obesity. The subject of the body composition analysis of nurses requires further research considering a more in-depth analysis of critical factors such as physical activity, stress level, diet, and shift work.

## Figures and Tables

**Table 1:** Characteristics of the study group in terms of age and parameters of the workplace.

Characteristic	*n*			%		
	M	Me	Min	Max	SD	CV
Age (years)	38.38	43	22	55	11.33	29.53
Age group						
Older	17			45.95		
Younger	20			54.05		
Seniority in years						
<2	9			24.32		
3-10	5			13.51		
11-20	3			8.11		
>20	20			54.05		
Working hours per week						
<30	1			2.70		
30-40	15			40.54		
40-50	15			40.54		
50-60	3			8.11		
>60	3			8.11		
Hours per day in a standing position						
<5	7			18.92		
5-8	15			40.54		
>8	15			40.54		

Abbreviations: M: mean; Me: median; Min: minimum; Max: maximum; SD: standard deviation; CV: coefficient of variation.

**Table 2:** Summary of body composition parameters in the study group.

Characteristic	M	Me	Min	Max	Q1	Q3	SD	CV
Height (cm)	165.8	166	156	180	162	169	5.6	3.4
Weight (kg)	66.4	63.7	49.6	104.2	59.9	70	11.6	17.5
BMI (kg/m^2^)	24.1	23.6	17.8	35.9	22.1	25.2	3.8	15.8
BF (%)	29.2	29.3	17.5	42.3	24.7	32.2	5.4	18.5
MM (%)	67.2	67.1	54.8	78.3	64.5	71.5	5.1	7.6
MA (years)	33.5	34	12	66	26	40	14.1	42.2
TBW (%)	50.6	50.4	41.2	59.4	48.5	53.7	4	7.9
ECW/TBW (rating)	43.2	43.2	39.6	47.1	42.2	44.3	1.8	4.1
VF (rating)	4.2	4	1	11	3	5	2.6	60.5

Abbreviations: BMI: body mass index; BF: body fat; MM: muscle mass; MA: metabolic age; TBW: total body water; ECW: extracellular water; VF: visceral fat; M: mean; Me: median; Min: minimum; Max: maximum; Q1: quartile 1^st^; Q3: quartile 3^rd^; SD: standard deviation; CV: coefficient of variation.

**Table 3:** Summary of body composition parameters depending on referral values.

Characteristic	Referral values	*n*	%
BMI (kg/m^2^)	Underweight	1	2.7
	Norm	26	7.3
	Overweight or obesity	10	27
BF (%)	Norm	29	78.4
	Too high	8	21.6
MM (%)	Norm	32	86.5
	Too high	5	13.5
MA (years)	Lower	27	73
	Higher	10	27
TBW (%)	Norm	34	91.9
	Lower	3	8.1
ECW/TBW (rating)	Norm	1	2.7
	Higher	36	97.3
VF (rating)^∗^	1	8	21.6
	2	1	2.7
	3	6	16.2
	4	5	13.5
	5	8	21.6
	6	5	12.5
	7	1	2.7
	8	1	2.7
	9	1	2.7
	10	1	2.7
	11	0	0

^∗^Table of cardinalities for particular values of VF rating. Abbreviations: BMI: body mass index; BF: body fat; MM: muscle mass; MA: metabolic age; TBW: total body water; ECW: extracellular water; VF: visceral fat; *n*: number of participants.

**Table 4:** Frequency of pain in the study group.

Localization	*n*	%	% answers
Cervical spine	23	62.2	17.8
Shoulders	17	46	13.2
Thoracic spine	21	56.8	16.3
Elbows	3	8.1	2.3
Wrists and/or hands	11	29.7	8.5
Lumbar spine	26	70.3	20.2
Hips and/or thighs	8	21.6	6.2
Elbows	14	37.8	10.9
Ankles and/or feet	6	16.2	4.7
Total	129	129	100

Abbreviation: *n*: number of participants.

## Data Availability

The data that support the findings of this study are available from the corresponding author upon reasonable request.
